# Prognostic Value of the Albumin-to-Prealbumin Ratio in Elderly Patients with Sepsis

**DOI:** 10.3390/jcm15051755

**Published:** 2026-02-25

**Authors:** Ichiro Hirayama, Minaho Nonaka, Hiromu Naraba, Tetsuhiro Yano, Mitsuru Ishii, Yoshiteru Tominaga

**Affiliations:** Department of Emergency Medicine, National Hospital Organization Saitama Hospital, Saitama 351-0102, Japan; nonaka374@gmail.com (M.N.); naraba-hok@umin.ac.jp (H.N.); teyano-tky@umin.ac.jp (T.Y.); 141mitsuru@gmail.com (M.I.); ganzytominaga@yahoo.co.jp (Y.T.)

**Keywords:** aged, inflammation, mortality, prealbumin

## Abstract

**Background**: Serum albumin and prealbumin are increasingly recognized as biomarkers of inflammation rather than nutritional status. Because prealbumin responds more rapidly to acute inflammatory and catabolic stress than albumin, the albumin-to-prealbumin ratio (APR) may better reflect inflammatory burden. However, its prognostic significance in elderly patients with sepsis remains unclear. **Methods**: This single-center retrospective observational study included 84 patients aged ≥75 years hospitalized with sepsis between April 2023 and March 2024. APR was calculated using serum albumin and prealbumin levels at admission, and patients were categorized into low- and high-APR groups based on the median value. The primary outcome was 28-day mortality. Multivariable logistic regression analysis was performed to evaluate the independent association between APR and 28-day mortality after adjustment for the Sequential Organ Failure Assessment score, maximum serum lactate level, and Mini Nutritional Assessment–Short Form. Subgroup analyses were conducted according to median serum albumin level (<2.9 vs. ≥2.9 g/dL). **Results**: Twenty-eight-day mortality was significantly higher in the high-APR group than in the low-APR group (40.5% vs. 7.1%, *p* = 0.001), with significantly poorer survival (log-rank *p* = 0.003). A high APR remained independently associated with increased 28-day mortality (odds ratio 10.2, 95% confidence interval 2.3–45.1). In subgroup analyses, APR was associated with mortality only among patients with relatively preserved albumin levels. **Conclusions**: APR is a useful prognostic marker of 28-day outcomes in elderly patients with sepsis and may provide prognostic information beyond conventional severity indices.

## 1. Introduction

Sepsis remains a major cause of morbidity and mortality worldwide, particularly among older adults [1]. Sepsis is characterized by a dysregulated host response to infection, resulting in profound systemic inflammation and metabolic stress [2]. With global population aging, the number of elderly patients affected by sepsis continues to rise, and this population accounts for a disproportionate share of sepsis-related deaths and healthcare utilization. Despite advances in intensive care management, the prognosis of elderly patients with sepsis remains poor, largely due to age-related vulnerability to systemic inflammation, metabolic stress, and organ dysfunction [3,4]. Accurate early risk stratification is therefore essential in this population; however, existing prognostic tools and biomarkers do not always adequately reflect the complex inflammatory and metabolic responses observed in older patients with sepsis [5]. Several inflammatory biomarkers, including C-reactive protein and leukocyte-derived indices, have been linked to adverse outcomes in sepsis [6,7,8]. However, these markers primarily reflect inflammatory activity and may not sufficiently capture the concurrent metabolic and catabolic alterations characteristic of severe sepsis, particularly in older adults.

Recent clinical guidelines have redefined the clinical interpretation of serum albumin and prealbumin levels, emphasizing their roles as biomarkers of inflammatory activity rather than as indicators of nutritional status [9]. During systemic inflammatory states, hepatic protein synthesis shifts toward acute-phase reactants, resulting in reduced production of constitutive proteins such as albumin and prealbumin [10]. Although both proteins decrease in response to systemic inflammation, their biological characteristics differ substantially. Prealbumin has a rapid turnover rate, with a half-life of approximately 48 h, and responds promptly to acute metabolic stress and accelerated protein catabolism [11]. In contrast, serum albumin reflects longer-term physiological and nutritional conditions over several weeks. Because of this shorter half-life, prealbumin concentrations decline more rapidly and markedly during acute inflammatory and catabolic stress. Greater reductions in prealbumin have been associated with adverse outcomes in critically ill populations [12].

In the setting of acute inflammation, prealbumin levels typically decrease more rapidly and to a greater extent than albumin levels, which can lead to a relative elevation of the albumin-to-prealbumin ratio (APR) as inflammatory severity increases. Accordingly, APR has been suggested as an integrated indicator reflecting both acute inflammatory activity and underlying metabolic stress. Furthermore, serum concentrations of both albumin and prealbumin are affected by intravascular volume status and hydration [13], potentially limiting their reliability when interpreted individually. By combining these two biomarkers into a single ratio, APR may offer a more robust representation of systemic inflammation and protein catabolic dynamics.

Previous studies have reported that elevated APR values are associated with increased inflammatory burden and adverse long-term outcomes in patients undergoing peritoneal dialysis and in other chronic disease populations [1,14]. However, evidence regarding the prognostic utility of APR in acute critical illness, especially sepsis, remains limited. Moreover, older adults with sepsis often exhibit reduced physiological reserves, age-associated frailty, diminished protein stores, and increased susceptibility to inflammation-induced suppression of hepatic protein synthesis [3]. These factors may amplify alterations in albumin and prealbumin dynamics, potentially enhancing the clinical relevance of APR in this vulnerable population. Conventional prognostic markers, including serum albumin, lactate levels, and severity scores, may not fully capture these age-specific inflammatory and metabolic alterations.

Although hypoalbuminemia and low prealbumin levels have individually been associated with poor outcomes in patients with sepsis, the clinical significance of APR as an integrated prognostic marker in elderly patients with sepsis has not been adequately investigated. To date, few studies have specifically examined whether APR provides incremental prognostic information beyond established severity indices in very old patients with sepsis.

We hypothesized that an elevated APR reflects heightened inflammatory burden and metabolic stress and is associated with worse clinical outcomes in elderly patients with sepsis. Accordingly, the aim of the present study was to evaluate the prognostic significance of APR in patients aged 75 years and older hospitalized with sepsis and to examine its association with short-term mortality. By clarifying the clinical relevance of APR, this study seeks to provide insight into a simple and readily available biomarker that may facilitate early risk stratification in this high-risk population.

## 2. Materials and Methods

### 2.1. Study Design and Participants

This retrospective observational study was conducted at a single tertiary emergency and critical care center in Japan. We reviewed the medical records of consecutive patients aged 75 years or older who were admitted to the hospital with a diagnosis of sepsis between 1 April 2023, and 31 March 2024.

Sepsis was defined according to the Sepsis-3 criteria as suspected or confirmed infection accompanied by an acute increase in the Sequential Organ Failure Assessment (SOFA) score of ≥2 points [1]. Eligible patients were identified from the electronic medical record system based on the attending physician’s diagnosis at admission and subsequently confirmed by chart review.

**Figure 1:**
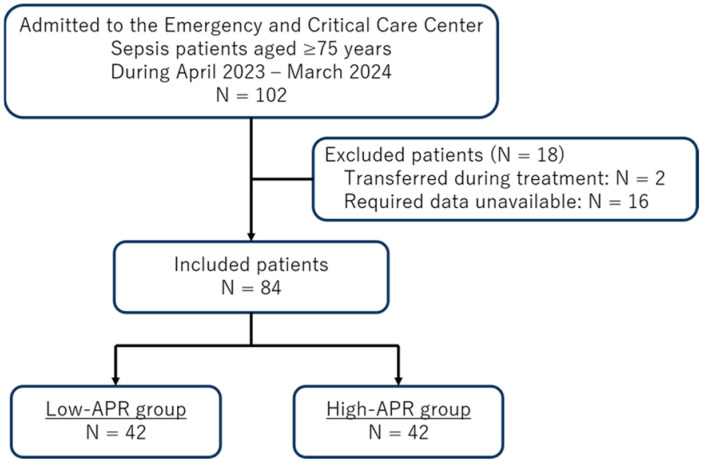
Flowchart of the study population. APR, albumin-to-prealbumin ratio.

A total of 102 patients met the initial inclusion criteria. We excluded two patients who were transferred to other institutions during hospitalization and 16 patients for whom essential clinical or laboratory data required for the analysis were unavailable. After these exclusions, 84 patients were included in the final study population (Figure 1).

The study protocol was conducted in accordance with the Declaration of Helsinki (2013 revision) and was approved by the institutional review board of our hospital (approval number: R2023-08). Given the retrospective nature of the study and the use of anonymized data, the requirement for written informed consent was waived in accordance with institutional regulations.

### 2.2. Data Collection

Clinical and demographic data were retrospectively extracted from electronic medical records by the study investigators using a standardized data collection form. Variables collected at the time of hospital admission included age, sex, height, weight, body mass index (BMI), comorbid conditions (hypertension, diabetes mellitus, cardiovascular disease, pulmonary disease, chronic kidney disease, and malignancy), use of vasopressors, and requirement for endotracheal intubation.

Laboratory data obtained at admission included serum albumin concentration, serum prealbumin concentration, white blood cell count, C-reactive protein level, and blood lactate level. All laboratory measurements were performed as part of routine clinical practice at the hospital’s central laboratory using standardized automated assays.

Disease severity was assessed using the Acute Physiology and Chronic Health Evaluation (APACHE) II score and the Sequential Organ Failure Assessment (SOFA) score, calculated based on the worst values recorded within the first 24 h after admission. Nutritional status and functional capacity were evaluated using the Mini Nutritional Assessment–Short Form (MNA–SF) and the Barthel Index, respectively, as documented at admission or within the first 48 h of hospitalization.

The albumin-to-prealbumin ratio (APR) was calculated by dividing the serum albumin concentration (g/dL) by the serum prealbumin concentration (mg/dL), both measured at the time of admission. To ensure unit consistency, prealbumin values were converted to g/dL prior to calculation when necessary. Patients were categorized into low-APR and high-APR groups based on the median APR value of the study cohort.

### 2.3. Outcome

The primary outcome was 28-day all-cause mortality following hospital admission. Survival status was determined through review of medical records.

### 2.4. Statistical Analysis

Continuous variables are presented as medians with interquartile ranges (IQRs), and categorical variables are summarized as frequencies and percentages. Comparisons between groups were performed using the chi-square test or Fisher’s exact test for categorical variables, as appropriate.

Survival analyses were conducted using the Kaplan–Meier method, and differences between groups were assessed using the log-rank test. In these analyses, APR category (low vs. high) was treated as the explanatory variable, and time from admission to death within 28 days was defined as the outcome variable. Patients who survived beyond 28 days were censored at that time point.

To evaluate the independent association between APR and 28-day mortality, multivariable logistic regression analysis was performed. Clinically relevant covariates were selected a priori based on previous literature and included SOFA score, maximum blood lactate level, and MNA–SF score. Results are reported as odds ratios (ORs) with 95% confidence intervals (CIs). Subgroup analyses were conducted by stratifying patients according to the median serum albumin concentration at admission (<2.9 g/dL vs. ≥2.9 g/dL), and APR values were compared between survivors and non-survivors within each subgroup.

All statistical analyses were performed using EZR (Saitama Medical Center, Jichi Medical University, Saitama, Japan), a graphical user interface for R software (version 4.2.2; R Foundation for Statistical Computing, Vienna, Austria). A two-tailed *p*-value of <0.05 was considered statistically significant.

## 3. Results

### 3.1. Patient Characteristics

A total of 84 patients aged 75 years or older with sepsis were included in the final analysis and were equally divided into the low-APR group (*n* = 42) and the high-APR group (*n* = 42) based on the median APR value. The baseline demographic, clinical, laboratory, and outcome characteristics are summarized in Table 1.

Patients in the high-APR group had a significantly lower body mass index and significantly higher SOFA scores at admission compared with those in the low-APR group. No significant differences were observed between the groups with respect to age, sex, source of infection, comorbidities, inflammatory markers (white blood cell count and C-reactive protein), blood lactate levels, use of vasopressors, requirement for endotracheal intubation, nutritional status assessed by the MNA-SF, or functional status assessed by the Barthel Index.

With regard to clinical outcomes, 28-day mortality was significantly higher in the high-APR group than in the low-APR group (40.5% vs. 7.1%, *p* = 0.001).

### 3.2. Comparison of 28-Day Prognosis

Twenty-eight-day mortality differed markedly between the two groups, with patients in the high-APR group exhibiting a significantly higher mortality rate than those in the low-APR group (40.5% vs. 7.1%, *p* = 0.001). Accordingly, 28-day survival was significantly lower in the high-APR group (59.5%) compared with the low-APR group (92.9%).

**Figure 2:**
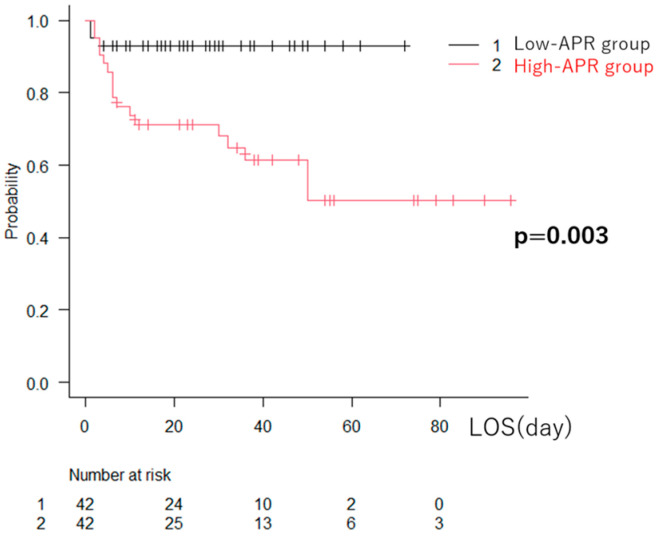
Kaplan–Meier curves for length of hospital stay stratified by albumin–prealbumin ratio (APR).

Kaplan–Meier survival analysis demonstrated a clear and early separation of the survival curves, indicating a significantly lower probability of survival over the 28-day follow-up period in the high-APR group (log-rank test, *p* = 0.003) (Figure 2).

In multivariable logistic regression analysis adjusting for clinically relevant prognostic factors, high APR remained independently associated with increased 28-day mortality (odds ratio [OR], 10.2; 95% confidence interval [CI], 2.3–45.1; *p* = 0.002). In contrast, SOFA score, maximum lactate level, and MNA-SF score were not independently associated with 28-day mortality in the adjusted model (Table 2).

The high-APR group showed a significantly lower survival probability compared with the low-APR group over the follow-up period. APR, albumin-to-prealbumin ratio; LOS, length of hospital stay.

### 3.3. Association Between APR and Survival Stratified by Serum Albumin Levels

To further evaluate the relationship between APR and prognosis, an exploratory subgroup analysis was conducted according to serum albumin levels at admission. Patients were stratified based on the median serum albumin concentration (2.9 g/dL) into a low-albumin group (<2.9 g/dL) and a preserved-albumin group (≥2.9 g/dL). The distribution of serum albumin and prealbumin levels according to 28-day survival status is shown in Figure S1.

**Figure 3:**
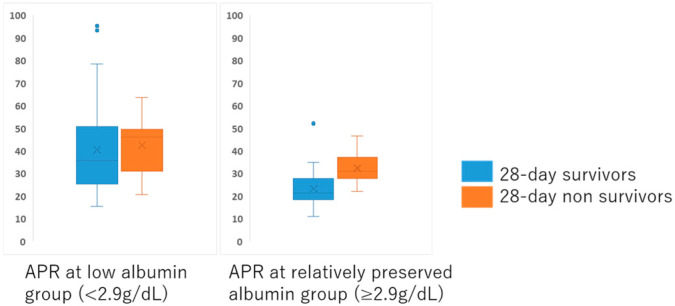
Albumin–prealbumin ratio (APR) in 28-day survivors and non-survivors stratified by serum albumin levels.

Within the low-albumin group, APR values did not differ significantly between survivors and non-survivors (median: 35.9 vs. 46.3, *p* = 0.350). In contrast, among patients with preserved serum albumin levels, APR values were significantly higher in non-survivors than in survivors (median: 31.1 vs. 21.5, *p* = 0.007) (Figure 3).

In patients with preserved albumin levels, APR was significantly higher in non-survivors than in survivors, whereas no significant difference was observed in the low-albumin group. APR, albumin-to-prealbumin ratio

## 4. Discussion

This study evaluated the prognostic significance of APR in patients aged 75 years and older hospitalized with sepsis.

### 4.1. Principal Findings

In this study, a higher APR was associated with significantly increased 28-day mortality in older patients with sepsis. This association remained significant in multivariable logistic regression analysis after adjustment for established prognostic factors, including SOFA score, maximum serum lactate level, and nutritional status assessed by MNA-SF. Although the confidence interval was wide, likely due to the limited sample size, the direction and magnitude of the association support the clinical relevance of APR as an independent prognostic marker. Importantly, this finding suggests that APR may be useful for prognostic assessment specifically in patients with relatively preserved serum albumin levels.

### 4.2. Comparison with Previous Studies

Previous studies have shown that decreased serum albumin and prealbumin levels are associated with increased mortality in patients with sepsis and in hospitalized older adults [15,16,17,18]. However, these biomarkers are strongly influenced by non-nutritional factors, including inflammatory burden, intravascular volume status, and comorbid conditions [17]. To overcome these limitations, APR has been proposed as a composite index that may partially account for such confounding effects. Consistent with this concept, higher APR values have been associated with poorer outcomes in patients undergoing peritoneal dialysis [14]. The present study extends these findings to older patients with sepsis and further demonstrates the prognostic value of APR after adjustment for disease severity and metabolic stress.

### 4.3. Pathophysiological Considerations and Clinical Implications

A key contribution of this study is the demonstration that APR may serve as a clinically meaningful prognostic indicator in older patients with sepsis. A high APR remained independently associated with increased 28-day mortality even after adjustment for established prognostic factors, including the SOFA score [19,20] and lactate level [21,22]. This finding suggests that APR reflects inflammatory and catabolic processes that are not fully captured by conventional severity indices. Although the confidence interval was wide, this was likely due to the limited sample size.

A disproportionate decline in prealbumin relative to albumin during severe systemic inflammation may contribute to an elevated APR. Such an imbalance may reflect heightened inflammatory burden and accelerated protein catabolism in vulnerable older patients.

In addition, the higher APR observed in patients with lower BMI may reflect reduced protein reserves and increased vulnerability to inflammation-induced suppression of prealbumin synthesis. Frail or undernourished individuals may experience a greater relative decline in prealbumin during acute inflammatory stress, thereby amplifying the APR. This interaction between nutritional vulnerability and systemic inflammation may partially explain the observed association between APR and mortality.

Both albumin and prealbumin concentrations are influenced by intravascular volume status and hydration [13]. By integrating these two parameters into a single ratio, APR may provide a more stable reflection of inflammatory burden than either marker alone. In the context of severe inflammation, a disproportionate decline in prealbumin relative to albumin may result in an elevated APR, thereby explaining its association with worse outcomes in older patients with sepsis.

The non-significant role of established predictors such as SOFA score and lactate level in the adjusted model should be interpreted cautiously. This finding may reflect limited statistical power or collinearity among severity-related variables rather than a lack of clinical importance.

Our findings further suggest that APR may provide incremental prognostic information primarily in patients with relatively preserved albumin levels, whereas its utility appears limited in those with marked hypoalbuminemia. In patients with severe hypoalbuminemia, albumin itself may reflect overwhelming inflammatory and catabolic stress, potentially diminishing the incremental prognostic contribution of APR.

### 4.4. Limitations

This study has some limitations. First, its retrospective single-center design may restrict the generalizability of the results and precludes causal inference. Although multivariable adjustment was performed, residual confounding cannot be excluded. Unmeasured factors such as pre-existing frailty, sarcopenia, malignancy burden, hepatic dysfunction, renal protein loss, fluid balance, and variations in treatment intensity may have influenced both APR levels and clinical outcomes. Second, we were unable to comprehensively assess comorbidities that affect protein metabolism, such as chronic liver disease, nephrotic syndrome, and protein-losing enteropathy. In addition, fluid resuscitation and hemodynamic management, which may alter intravascular protein concentrations through dilutional effects, were not systematically evaluated. Although lower BMI was associated with higher APR in our cohort, we did not formally evaluate the interaction between BMI, frailty status, and inflammatory burden. Third, APR was determined only at the time of admission, and changes during the clinical course were not evaluated. Serial measurements may provide additional prognostic information and warrant further investigation. Future multicenter prospective studies focusing on older populations are required to confirm these findings and determine whether serial APR determinations further enhance prognostic accuracy. Moreover, the incremental predictive value of APR relative to established risk markers requires formal validation in larger prospective multicenter cohorts, including younger populations, before routine clinical application can be considered.

## 5. Conclusions

In patients aged 75 years and older with sepsis, APR was independently associated with 28-day mortality and may be a useful prognostic marker of short-term outcomes, particularly among those with relatively preserved serum albumin levels. However, these findings should be considered hypothesis-generating, and confirmation in larger prospective multicenter studies is required before routine clinical application can be recommended.

## Figures and Tables

**Table 1:** Characteristics of patients aged ≥75 years with sepsis.

		Low-APR Group *n* = 42		High-APR Group *n* = 42		*p*-Value
Age, y		86.5	81.3–90.8	86	81.0–91.0	0.760
Male sex		23	54.8	23	54.8	1.000
BMI, kg/m^2^		20.7	19.1–24.5	19.2	17.0–22.0	0.033
Source of infection	Pneumonia	23	54.8	25	59.5	0.826
	Urinary tract infection	13	31.0	14	33.3	1.000
	Others	6	14.3	3	7.1	0.483
Patient history	Hypertension	28	66.7	24	57.1	0.501
	Diabetes mellitus	15	35.7	14	33.3	1.000
	Cardiovascular disease	17	40.5	17	40.5	1.000
	Pulmonary disease	7	16.7	7	16.7	1.000
	CKD	5	11.9	0	0.0	0.055
	Cancer	5	11.9	12	28.6	0.101
Blood test data	WBC, ×10^3^/mm^3^	11.4	9.0–16.0	13.0	7.4–17.1	0.961
	CRP, mg/dL	6.54	2.75–11.94	14.19	4.73–21.80	0.070
	lactate, mmol/L	3.0	1.8–5.0	3.2	2.3–5.2	0.431
Clinical severity	APACHEII score	16.5	13.0–22.0	21	15.0–26.8	0.076
	SOFA score	5	3.3–8.0	8	6.0–10.8	0.014
Use of vasopressors		17	40.5	23	54.8	0.275
Requirement for endotracheal intubation		3	7.1	6	14.3	0.483
Nutritional assessment	MNA-SF	9	7.0–12.0	8.5	6.3–10.0	0.122
ADL	Barthel index	50	10.0–95.0	85	41.3–100.0	0.094
Outcome	28-day mortality	3	7.1	17	40.5	0.001

Summary statistics are reported as No. (%) or medians (interquartile range). ADL, Activities of Daily Living; APACHE II, Acute Physiology and Chronic Health Evaluation II; APR, albumin-to-prealbumin ratio; BMI, body mass index; CKD, chronic kidney disease; CRP, C-reactive protein; MNA-SF, Mini Nutritional Assessment—Short Form; SOFA, Sequential Organ Failure Assessment; WBC, white blood cell count.

**Table 2:** Multivariable Logistic Regression Analysis for 28-Day Mortality.

Variable	OR	95%CI	*p*-Value
High-APR group (ref: Low APR)	10.2	2.3–45.1	0.002
SOFA score (per 1 point increase)	1.16	0.968–1.39	0.108
Max lactate (per 1 mmol/L increase)	1.11	0.914–1.35	0.289
MNA-SF (per 1 point increase)	1.13	0.907–1.41	0.276

APR, Albumin-to-Prealbumin Ratio; CI, Confidence Interval; OR, Odds Ratio; SOFA, Sequential Organ Failure Assessment; MNA-SF, Mini Nutritional Assessment—Short Form.

## Data Availability

The datasets used and/or analyzed in this study are available from the corresponding author upon reasonable request.

## Supplementary Materials

The following supporting information can be downloaded at: https://www.mdpi.com/article/10.3390/jcm15051755/s1, Figure S1: Scatter plot comparing serum albumin and prealbumin levels between 28-day survivors and non-survivors.

## Abbreviations

APACHE	Acute Physiology and Chronic Health Evaluation
APR	Albumin-to-Prealbumin Ratio
MNA–SF	Mini Nutritional Assessment—Short Form
SOFA	Sequential Organ Failure Assessment
