# Supplementary material for: Prognostic Value of the Albumin-to-Prealbumin Ratio in Elderly Patients with Sepsis
Source: J Clin Med. 2026 Feb 25;15(5):1755. doi: 10.3390/jcm15051755 (PMC12986160; doi:10.3390/jcm15051755)
Supplement: Supplementary file 1 [file jcm-15-01755-s001.zip › jcm-4144613-supplementary.pptx]

## Slide 1
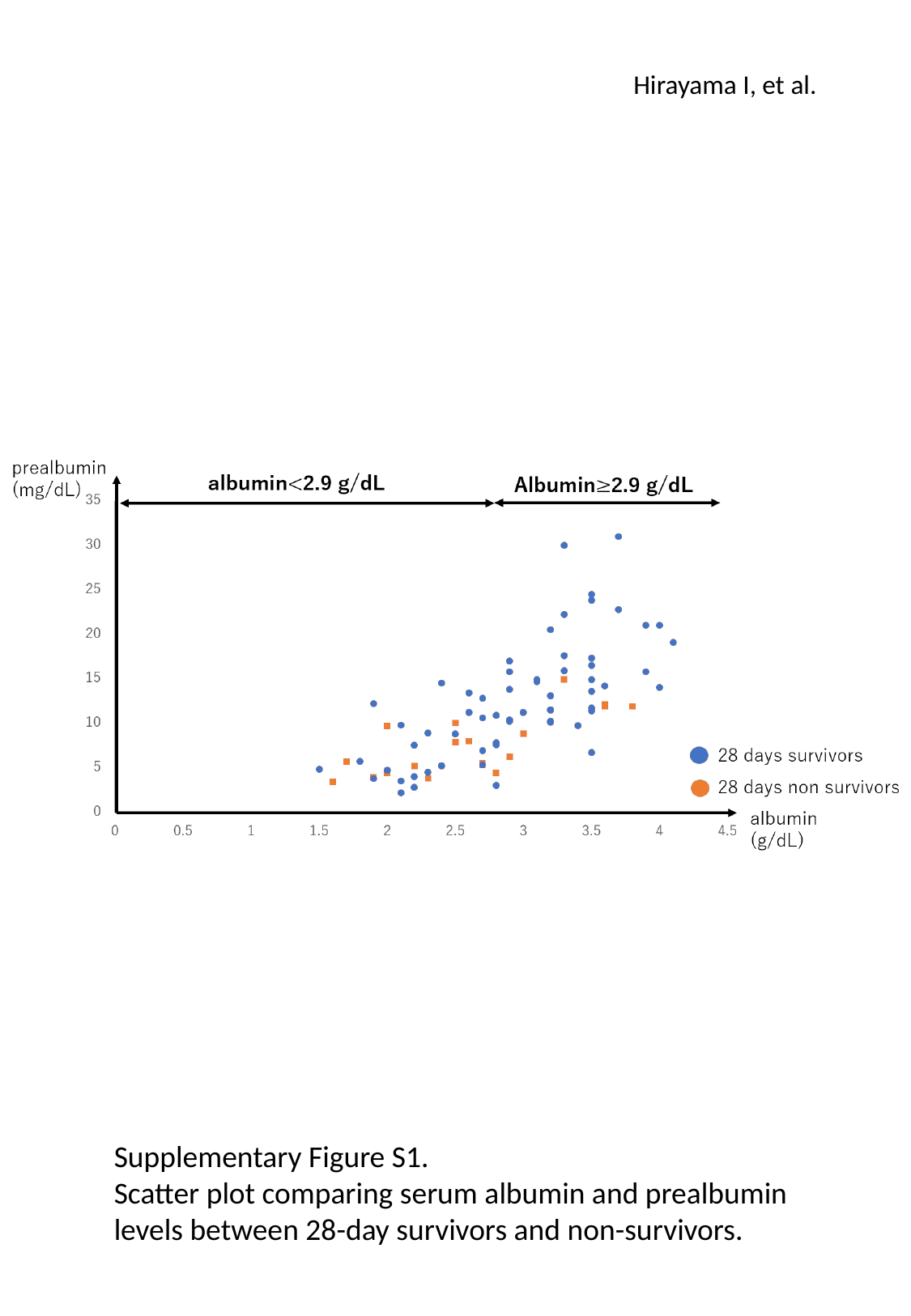

Hirayama I, et al.
Supplementary Figure S1.
Scatter plot comparing serum albumin and prealbumin levels between 28-day survivors and non-survivors.
